# *Litchi chinensis* as a Functional Food and a Source of Antitumor Compounds: An Overview and a Description of Biochemical Pathways

**DOI:** 10.3390/nu9090992

**Published:** 2017-09-08

**Authors:** Sonia Emanuele, Marianna Lauricella, Giuseppe Calvaruso, Antonella D’Anneo, Michela Giuliano

**Affiliations:** 1 Department of Experimental Biomedicine and Clinical Neurosciences, Laboratory of Biochemistry, University of Palermo, 90127 Palermo, Italy; sonia.emanuele@unipa.it (S.E.); marianna.lauricella@unipa.it (M.L.); 2 Department of Biological, Chemical and Pharmaceutical Sciences and Technologies, Laboratory of Biochemistry, University of Palermo, 90127 Palermo, Italy; giuseppe.calvaruso@unipa.it (G.C.); antonella.danneo@unipa.it (A.D.)

**Keywords:** *Litchi chinensis* fruit extracts, nutraceutical properties, antitumor activity

## Abstract

Litchi is a tasty fruit that is commercially grown for food consumption and nutritional benefits in various parts of the world. Due to its biological activities, the fruit is becoming increasingly known and deserves attention not only for its edible part, the pulp, but also for its peel and seed that contain beneficial substances with antioxidant, cancer preventive, antimicrobial, and anti-inflammatory functions. Although literature demonstrates the biological activity of Litchi components in reducing tumor cell viability in *in vitro* or *in vivo* models, data about the biochemical mechanisms responsible for these effects are quite fragmentary. This review specifically describes, in a comprehensive analysis, the antitumor properties of the different parts of Litchi and highlights the main biochemical mechanisms involved.

## 1. Introduction

*Litchi chinensis* Sonnerat (also known as Chinese cherry, lychee nut, leechee, etc.) is a fruit tree belonging to Sapindaceae family. It was originally cultivated in China for more than 2300 years and in northern Vietnam. Some cultivars in the west of Guangdong region in China have a long history of cultivation, while others are relatively new. It has been reported that cultivars such as Sum Yee Hong, Haak Yip, Kwai May, No Mai Chee, Wai Chee, and Seong Sue Wai date back to 500 or 600 years or more, while others, such as Bah Lup, Heong Lai and Tim Naan, or Souey Tung are relatively young cultivars (about 100–300 years old) [1,2,3].

Traditionally, Litchi cultivar characterization was based on morphological studies focused on traits such as floral and fruit characteristics and the harvest season [4]. However, despite Litchi cultivation being diffused in many tropical and sub-tropical regions in the world, this approach is limited because it does not evaluate the interaction of morphological traits with environmental conditions [5]. Only recently, a study was undertaken to identify Litchi cultivars and their genetic relationships using new generation molecular approaches such as single nucleotide polymorphism (SNP) markers [6]. This strategy has allowed for cultivar standardization nomenclature facilitating the integration and interpretation of Litchi germoplasm.

Today, Litchi is mainly present in many countries of Southeast Asia, the Indian subcontinent, and South Africa, and in other tropical or sub-tropical areas around the world even if China, India, Thailand, and Vietnam are the leading Litchi-producing countries in the world. Recently, its cultivation, together with other sub-tropical orchards [7], has been also launched in Southern Europe, such as in Spain and more recently in Sicily, a Southern Italian island, where the climatic conditions (cold winter, especially at the beginning of flowering and the hot humid climate during the growth of the fruit) have been proved to be favorable for this crop. In Sicily region the most diffused cultivars are Wai-Chee, Kwai May, and Mclaine [8].

**Figure 1:**
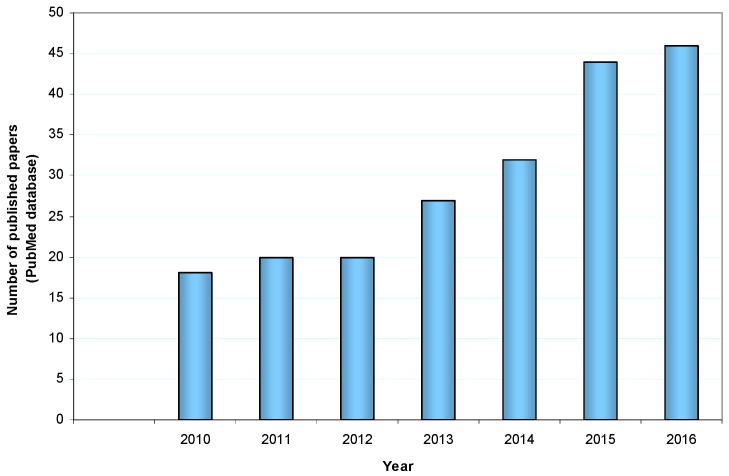
Number of papers on *Litchi chinensis* biological activities published in the last seven years (font PubMed database, https://www.ncbi.nlm.nih.gov/pubmed).

The interest for this crop is associated to the goodness of its fruits and the high nutritional value. Investigations demonstrated a long series of beneficial health compounds including antioxidant, cancer preventive, antimicrobial, anti-inflammatory activities, and so on, so that in 2012 *Litchi chinensis* was inserted in the list of functional foods [9]. In parallel to the identification of the bioactive components of Litchi fruit (but also of the other portions of the plant), in recent years the number of published articles showing the different biological activities of Litchi components has greatly increased (Figure 1).

Beyond their healthy potential, some studies have also indicated that some parts of the fruit could find application in other commercial sectors. Discarded fruit peels can prove to be good source for dyeing textile material, a sort of application that may have a great commercial impact. Litchi peel extract has also been proven to act as a potential green inhibitor in the corrosion of mild steel [10].

## 2. The Plant

**Figure 2:**
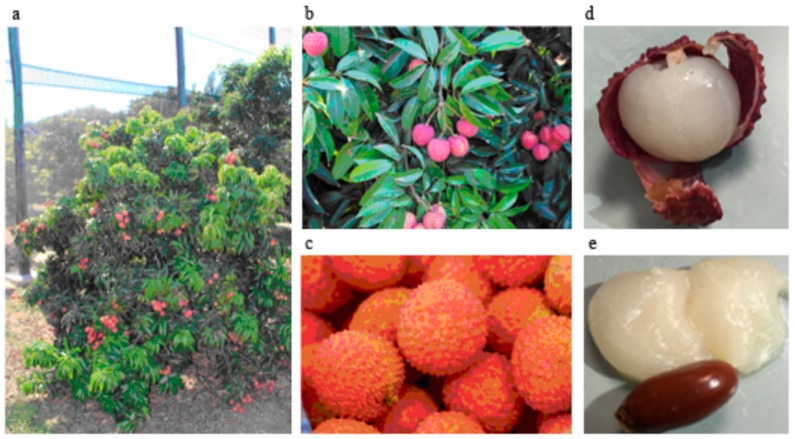
Sicilian *Litchi chinensis* tree, Kway May cultivar (**a**), Sicilian Litchi fruit. Kway May (**b**) and Way Chee (**c**) cultivars. The details of the pulp and seed are reported in (**d**,**e**), respectively (with the permission of Azienda Siciliana Cupitur, Caronia, Sicily).

*Litchi chinensis* is an arboreal and evergreen tree; the bark is grey-black, the branches present shiny, lanceolate leaves of deep green color, and a brownish-red, dense and rounded-shape crown, which can be very large, up to 15–20 m in origin countries, while in other areas, such as in Sicily, comes to 6–8 m in height (Figure 2a). Flowers, which grow on a terminal inflorescence, are small, without corolla, and yellowish-white. Litchi fruits, which are similar in volume to a strawberry, are in pendulous clusters, roundish, green, and, once mature, become pinkish or reddish (Figure 2b,c). The fruits of Litchi have a thin and rigid peel that easily comes off to show a pearl-white jelly-like pulp with excellent flavor due to the combination of acids and sugars (Figure 2d,e). In tropical countries, the fruit reaches the maturation in the late autumn, while in Sicily it ripens in August.

Litchi fruit is rich in carbohydrates and fibers while lipids and proteins are scarce (Table 1). It is also appreciated for its nutritional properties: in fact the fruit is very rich in nutraceuticals, fundamental compounds with extra health benefits in addition to the basic nutritional value found in foods. Beyond the differences between cultivars of various origins, the beneficial effects of fruits have been partly related to their high contents in micronutrients including vitamins (B1, B2, B3, B6, C, E, K), carotenoids, minerals (potassium, copper, iron, magnesium, phosphorus, calcium, sodium, zinc, manganese, and selenium), and polyphenols (Table 1), which are highly represented in comparison with other tropical fruits [11,12].

Nutritive value and the amounts of macrocomponents, referring to fresh fruit, are from the National Nutrient Database for Standard Reference (United States Department of Agriculture, Washington, DC, USA) [13], while those concerning the microcomponent amounts are from Septembre-Malaterre [12].

Studies evidenced that Litchi fruit from different cultivars may show significant qualitative and/or quantitative differences in their composition. By analyzing the total content of phenolics, flavonoids, anthocyanins, and procyanidins in the pericarp of nine commercially available Litchi cultivars, Li et al. [14] found a 3.2-fold difference in phenolic content between the highest and lowest Litchi varieties, Heiye and Chanchutou, respectively. Similar results were obtained comparing the levels of flavonoids and anthocyanins, while no difference seemed to be in the individual procyanidin composition of the different Litchi varieties analyzed. These results indicate that the significant differences in phytochemical profiles among the varieties should be considered for their potential application to promote health.

## 3. Litchi Fruit as a Functional Food

In recent years, medicine has started to pay great attention to functional food, which displays an additional function related to health promotion or disease prevention. Since it is well known that particular dietary factors and lifestyle can promote cancer development, research is increasingly betting on some protective components of vegetables and fruits or phytocompounds enriched with antitumor properties that can exert cancer prevention or even antitumor properties. The tumor-protective effects of dietary factors are most likely mediated by multiple signaling pathways, consistent with the heterogeneous nature of the disease and the distinct genetic profiles of different tumors. The effects induced by molecules with antitumor activity are often related to cell cycle arresting ability or tumor targeting pro-apoptotic action which might be dependent on unbalanced redox equilibrium. Even though the relationship between the redox state in tumor cells and anticancer response is quite complicated and sometimes controversial, it is known that some agents, that are capable of inducing oxidative stress, can promote tumor cell death [15]. On the other hand, there is ample evidence that phytochemicals with antioxidant properties can behave as potent antitumor agents [16,17]. In this scenario, Litchi fruit is currently emerging as a potential functional food due to its important nutraceutical properties and chemical composition, which includes specific components endowed with antioxidant as well as anticancer activities. It is important to emphasize that not only the pulp, which is the only edible part of the fruit, contains bioactive compounds that can exert anti-proliferative effects in tumor cells, but also the peel and the seed are enriched with substances that are potentially beneficial and endowed with antitumor properties. Their isolation and characterization can thus open up the possibility to produce novel active principles with a potential application in cancer therapy. 

Recently, Ibrahim and Mohamed [18] exhaustively reviewed chemical constituents and pharmacological activities of *Litchi chinensis*, listing the single constituents identified over the past few decades. Here, among the multiple biological activities of the different portions of the Litchi fruit, we specifically highlight the major findings in the literature on the anticancer properties. In particular, the next sections of this review describe the composition of the different parts of Litchi fruit and report the effects of their components in different *in vitro* and *in vivo* tumor models. It has to be considered, however, that in many papers antitumor properties have been attributed to Litchi crude extracts of the different portions and only in some cases to single biochemical compounds that have been isolated and characterized. Moreover, a certain part of the literature only refers to anti-proliferative effects of Litchi extracts but poorly describes the biochemical mechanisms involved. We thus made an effort to critically summarize in this review the most significant contributions to the elucidation of the biochemical effects induced by particular components of the Litchi portions.

## 4. Antitumor Properties of Litchi Pulp-Derived Components 

The fresh Litchi fruit has a delicate whitish pulp with a floral smell and a fragrant, sweet flavor. This portion is enriched with a blend of components with interesting nutraceutical properties. Specifically, many studies have shown that Litchi pulp contains a great percentage of bioactive polysaccharides with strong antioxidant activity [19,20,21]. In addition, these compounds display antitumor and immunomodulatory effects *in vitro* [22]. Specifically, as regards the anti-proliferative effects of polysaccharides contained in the pulp fraction, these authors assert that dried Litchi pulp, which contains more proteins, uronic acid, arabinose, galactose, and xylose, exerts greater effects than the fresh pulp in different tumor cell lines, including hepatocarcinoma HepG2, HeLa, and lung adenocarcinoma A549 cells. In addition, dried Litchi pulp was shown to be a better stimulator of spleen lymphocyte proliferation, NK cell cytotoxicity, and macrophage phagocytosis. The authors thus concluded that drying enhances the bioactivity of polysaccharides contained in Litchi pulp [22]. Moreover, they have shown that the anti-proliferative effect of Litchi pulp in tumor cells was higher when extraction of polysaccharides was carried out with 80% ethanol, which contained more galactose and mannose compared to the fractions obtained with 40% and 60% ethanol solutions [23]. Immunomodulatory and antioxidant effects of Litchi pulp polysaccharides have been also described *in vivo* in a cyclophosphamide-induced immunosuppression model by the same authors [21].

The antioxidant and anti-tumor activity of Litchi pulp extracts has been attributed not only to polysaccharides but also to bioactive phenolic compounds, in particular polyphenols, a structural class of natural, organic chemicals characterized by the presence of multiple phenol structural units. These compounds are abundant micronutrients with antioxidant properties in our diet, and evidence is emerging for their role in cancer prevention [24]. Zhang et al. [25] have detected six individual phenolics (gallic acid, chlorogenic acid, (+)-catechin, caffeic acid, (−)-epicatechin, and rutin) in litchi pulp by high-performance liquid chromatography (HPLC), and reported their antioxidant activity by Frap assay. 

More recently, analysis by reverse-phase preparative HPLC has revealed that the three-polyphenol components with major antioxidant activity in Litchi pulp fraction were quercetin 3-rut-7-rha, rutin, and epicatechin [26]. Phenolic-rich Litchi pulp extracts administered at a dosage of 200 mg/kg/day for 3 consecutive weeks have been also shown to protect *in vivo* the liver against restraint stress-induced damage by increasing the activity of free radicals scavenger enzymes (glutathione peroxidase, superoxide dismutase, catalase) and reducing mitochondrial reactive oxygen species (ROS) production [27].

A wide literature refers to the anticancer properties of natural catechins [28,29] and in particular epigallocatechin has been used as a potent chemopreventive agent [30,31]. Also, rutin has revealed antitumor properties; inhibiting proliferation, attenuating superoxide anion production, and affecting migration of human cancer cells, as reported by Ben Sghaier et al. [32]. From a biochemical point of view, both cathechins and rutin given at different doses (0.02%, 0.04%, 0.08%, 0.16%, or 0.32%) for 6 weeks as the sole source of drinking fluid to tumor-bearing mice increased the levels of E-cadherin on the plasma membrane, and decreased those of nuclear β-catenin, c-Myc, phospho-AKT, and phospho-ERK1/2 in the tumors [33]. Moreover, epigallocatechin-3-gallate has been shown to decrease IGF1 and restore IGF binding protein 3 (IGFBP3) levels in hepatocellular carcinoma cells. The modulation of IGF1/IGFR3 axis, which plays a critical role in the development of hepatocarcinoma, was associated with reduced levels of phosphoinositide 3-kinase (PI3K) as well as phospho-AKT and phospho-ERK1/2 [34]. In addition, gallic acid, a bioactive phenol compound present in the pulp, has been shown to induce apoptosis in tumor cells by reducing the activation of EGFR, ERK1/2, and AKT proteins and downregulating the expression of Cyclin D and Bcl-2 genes [35]. 

These studies and many others not cited here demonstrate that the dietary consumption of the fruit might be of great benefit. Further studies are needed to highlight the anti-tumor molecular mechanisms induced by isolated components of the Litchi pulp. In this regard, we are now carrying out research on the specific components of Litchi pulp, which behave as anti-tumor and pro-apoptotic agents in *in vitro* cancer models. 

## 5. Antitumor Properties of Litchi Peel-Derived Components

Litchi peel (pericarp), although not edible, is an important portion of the fruit that can be considered as a source of biologically interesting compounds. In particular, the pericarp has been shown to contain bioactive flavonoids and anthocyanins. The major flavonoids contained in this portion of the fruit are proanthocyanidin B2, proanthocyanidin B4, and epicatechin, while cyanindin-3-rutinside, cyanidin-3-glucoside, quercetin-3-rutinoside, and quercetin-3-glucoside represent important anthocyanins isolated by Litchi pericarp [36].

Both flavonoids and anthocyanins display antioxidant properties and can exert anticancer effects. Clear evidence of the antitumor action of Litchi pericarp extracts was obtained in human breast cancer cells where oligonucleotide microarray analysis revealed the upregulation of genes predominantly involved in cell cycle regulation, apoptosis, and signal transduction and the downregulation of those mainly associated with invasion and malignancy of cancer cells [37]. The same authors evidenced a significant tumor mass reduction *in vivo* using breast cancer mouse xenograft treated for 10 weeks with 0.3 mg/mL of Litchi fruit pericarp extracts; the effects were accompanied with remarkable increase of caspase-3 protein expression [37]. However, the results reported by Wang were obtained using a water-soluble ethanolic extract of Litchi fruit pericarp without the identification of the specific components. Successively, Zhao et al. [38] evidenced anti-breast cancer activities of epicatechin, proanthocyanidin B2, proanthocyanidin B4 extracted by Litchi pericarp together with immunomodulatory properties examined by evaluating proliferation of mouse splenocytes. Interestingly, epicatechin and proanthocyanidin B2 have been shown to display lower cytotoxicity in human breast cancer MCF-7 cells and human embryonic lung fibroblasts than paclitaxel [38]. A limit of this research, as well as in other cases, is that the anticancer activity is only reported as cell viability reduction measured by MTT assay and no biochemical mechanism is proposed. 

Although the literature referring to the potential antitumor properties of Litchi pericarp is quite dated, we believe that this part of the fruit represents a proper material to extract active principles that can be used in cancer research. This consideration is based on our current research that indicates a potent antitumor action of Litchi pericarp extracts. 

## 6. Antitumor Properties and Biochemical Aspects of Litchi Seed-Derived Components

The seed (endocarp) is a non-edible part of Litchi fruit, rather it can even be toxic to humans. The toxicity of Litchi seed is most likely due to the presence of methylene cyclopropyl-alanine (MCPA), also known as hypoglycin A, and its analogue methylene cyclopropyl-glycine (MCPG), both toxins causing hypoglycaemic encephalopathy [39,40]. Despite potential toxicity, Litchi seed extracts are widely used in Chinese popular medicine to relieve pain in different diseases, and there is increasing evidence that some components of this portion possess multiple activities, such as modulation of blood glucose and lowering of blood lipids [41,42,43], preventing liver injury [44], and exerting anti-oxidative and antiviral effects [45]. There is literature that refers to the potent antitumor properties exerted by Litchi seed extracts or specific isolated components from this portion. This paragraph critically summarizes the recent findings on this subject. 

Xiao et al. [46] evaluated the anticancer effects of Litchi seed extracts *in vivo* and provided the first evidence of the anti-tumor action of this portion of the fruit. Subsequently, other authors have focused on human hepatocellular carcinoma HepG2 cells, demonstrating reduced proliferation and the appearance of apoptotic morphological features following treatment with Litchi seed preparation [47]. More recently, Hsu et al. [48] provided evidence that seed extracts (25–50 μg/mL) affect the levels of cyclins determining G2/M cell cycle arrest, and induce a canonic apoptotic pathway which is accompanied with upregulation of the pro-apoptotic Bax protein and caspase-3 activation with PARP cleavage. In this study, the authors used hydro-alcoholic Litchi seed extracts and attribute the effects observed to polyphenols. Their analysis, in fact, identified the extract as polyphenol-rich fraction with flavonoids and condensed tannins as dominant components [48]. 

Actually, since Litchi seed contain a complex blend of components, it remains to be elucidated which specific compounds are endowed with antitumor properties. In this regard, Lin N. et al. [49] have shown that saponins, a class of terpenic glycosides extracted from the Litchi seed, inhibit hyperplasia of mammary gland tissue and influence estrogen-mediated signaling pathways in rats at the dose of 0.1 g/kg and 0.2 g/kg, respectively. More recently, these molecules have been proven to improve the cognitive function preventing neuronal injury [50]. Moreover, it has been shown that specific flavonoid glycosides purified from Litchi seeds, including litchioside D, taxifolin-glucopyranoside, and kaempferol neohesperidoside, exert strong anti-proliferative effects in different tumor cell lines including hepatocellular carcinoma HepG2, cervix cancer HeLa, lung carcinoma A549, and LAC cells [51]. 

With regard to the biochemical antitumor mechanisms induced by Litchi seed, most of the papers present in the literature refer to crude extracts of this portion. Lin et al. [52] showed that Litchi seed extracts regulate the ratio between Bcl-2 and Bax (two proteins of bcl-2 family with anti- and pro-apoptotic action, respectively) both *in vitro* and *in vivo* tumor models. More recent evidences have been provided that Litchi seed extracts (25–150 μg/mL) induce cell cycle arrest and apoptosis, suppressing cyclins and Bcl-2 and elevating the levels of Kip1/p27, Bax, and caspase -8, -9 and -3 activities in non-small cell lung carcinoma cells [53]. In the same paper, the authors showed that seed extracts inhibit epidermal growth factor receptor (EGFR) and its downstream Akt and Erk1/2 signaling.

In line with these observations, recently, in an elegant study, Guo et al. [54] demonstrated the effects of Litchi seed n-butyl alcohol extracts (60–120 μg/mL) in in vitro and *in vivo* models of prostate cancer. The authors analyzed in detail the proteins involved in cell proliferation and apoptosis as well as those involved in migration and invasion. They demonstrated activation of mitochondrial caspase-dependent apoptotic cascades, up-regulation of cyclin-dependent kinase (CDK) inhibitors, and inhibition of the correlated cyclin/CDK network and correlated the increased expression of E-cadherin, β-catenin, vimentin, and snail with the decrease in cell migration and invasion potential. The research on oncogenic signaling cascades underlying the observed effects is noteworthy. In fact, the authors identified the Akt/GSK3β signaling pathway as the target of Litchi seed extracts [49]. However, although the study analyzes new aspects of Litchi seed extract action, in our opinion, the direct involvement of this pathway should be better clarified. 

Based on our specific background regarding programmed cell death activated by different compounds in tumor cell lines [55,56,57,58,59,60], we have recently analyzed the effects of Sicilian Litchi fruit extracts in our experimental tumor models. This project, in collaboration with the “Sicilian local action group” (GAL) from “Castellammare”, aims to prompt cultivation and consumption of Litchi fruit in the Sicilian territory. In addition, it aims to consider the sustainability of using non-edible Litchi parts (seed and peel, at present, a waste product from Litchi processing) as a source of anti-tumor compounds. 

## 7. Other Parts of the Litchi Plant That Contain Antitumor Compounds

Other parts of Litchi have been used to extract antitumor compounds. For instance, Litchi leaf, which is a good resource of phenolics as well, can be considered a part of the plant with potential anticancer properties. In this regard, Wen et al. [61] extracted three phenolic compounds among which cinnamtannin B1 showed antiproliferative and antioxidant activities in tumor cells. In addition, new δ-tocotrienols and meroditerpene chromane were isolated from the leaves of Litchi and tested against human gastric adenocarcinoma and hepatocarcinoma cell lines [62]. 

Also, the Litchi flower contains bioactive compounds some of which have been shown to inhibit lipopolysaccharide-induced expression of pro-inflammatory mediators [63,64]. The analysis of inhibitory effects on lipopolysaccharide-induced expression of pro-inflammatory mediators induced by Litchi flower showed the involvement of NF-κB, ERK, and JAK2/STAT3 pathways in tumor cells [64].

Overall, the list of biologically active compounds isolated from *Litchi chinensis* is very long. The *in vitro* analysis of IC_50_ values showed that many of these components display a similar or even higher antitumor efficacy than chemotherapeutics used as positive controls. Moreover, some classes of compounds are noteworthy, such as chromanes extracted from Litchi leaves, which exhibited much lower IC_50_ values than 5-fluorouracil (IC_50_ 62.9 μM), a well-known chemotherapeutic drug [62]. Therefore, studies aimed at the identification of specific compounds with high antitumor efficacy could pave the way to a new generation of chemotherapeutics.

## 8. Oligonol

Among the Litchi components, Oligonol deserves particular attention. Oligonol is a polyphenol-rich Litchi extract processed to convert the high-molecular weight proanthocyanidins into low-molecular compounds to improve bioavailability [65,66]. Oligonol has shown favorable effects on various chronic diseases [67,68]. It has been shown to display an ameliorative effect on diabetes-induced alterations and renal disorders associated with gluco-lipotoxicity-mediated oxidative stress, inflammation, and apoptosis in type 2 diabetic db/db mice [66]. These effects could be ascribed to proanthocyanidins which have been reported to exhibit beneficial bioactivities in many studies [69]. In addition, some papers attribute antitumor properties to Oligonol [70,71]. In this regard, Yum et al. [72] have shown that Oligonol can behave as a tumor preventive agent since it inhibited azoxymethane-initiated and dextran sulfate sodium-promoted adenoma formation in the mouse colon. 

Specifically, the induction of apoptosis by Oligonol observed in MCF7 and MDA-MB-231 breast cancer cells is related to the regulation of Bcl-2 family members and the inactivation of ERK/MEK signaling [73]. Moreover, inhibitory effects of Oligonol on phorbol ester-induced tumor promotion and COX-2 expression have been reported in mouse skin and are associated with reduced NF-κB and C/EBP DNA binding [71]. Oligonol has been also shown to inhibit melanoma-derived lung metastasis in mice [70]. More recently, it has been shown that Oligonol treatment attenuates the production of inflammatory mediators and suppresses NF-κB and ERK phosphorylation in HepG2 cells and in *in vivo* models [74].

**Figure 3:**
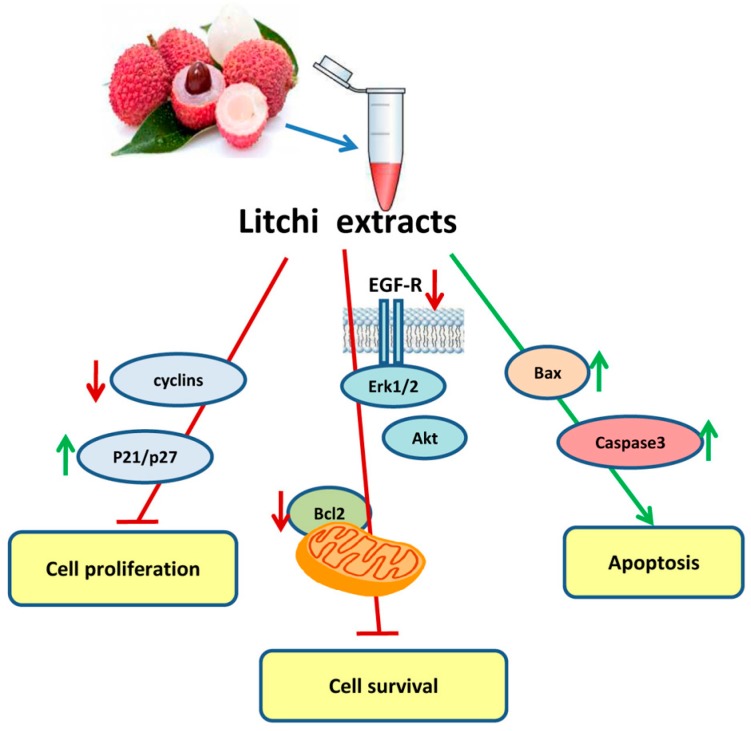
Scheme of the effects of Litchi extracts on cell proliferation, cell survival, and apoptosis. Red arrows indicate downregulated factors or processes, and green arrows indicate increased factors or processes.

Overall, the main biochemical mechanisms induced by Litchi extracts involved in anticancer effects are summarized in Figure 3 and described in Table 2.

## 9. Conclusions

The different portions of Litchi fruit contain key bioactive compounds that account for the anticancer effects described in the present review. Purifying these agents may represent an important step in phyto-pharmacotherapy, which can have a high impact in oncology. However, the biological activity of Litchi components has been mainly studied as evaluation of cytotoxicity in *in vitro* models. Therefore, the knowledge of the biochemical mechanisms underlying the anti-proliferative/death effects of Litchi components in tumor cells represents an important basis for anticancer translational studies. 

## Figures and Tables

**Table 1:** Principal macronutrients and micronutrients of Litchi fruit.

CALORIES	66 kcal/100 g
MACROCOMPONENTS (g/100 g)	
Carbohydrates	16.53
Lipid	0.44
Protein	0.83
Dietary fiber	1.30
Water	81.76
MICROCOMPONENTS	
Total carotenoid content (μg beta-carotene equivalent/100 g)	571.4 ± 117.2
Vitamin C content (mg ascorbic acid equivalent/100 g)	10.1 ± 2.2
Total polyphenol content (mg gallic acid equivalent/100 g)	178.0 ± 34.7
Total flavonoid content (mg quercetin equivalent/100 g)	53.3 ± 5.9

**Table 2:** Specific antitumor effects of Litchi fruit portions and principal involved biochemical pathways.

		Cancer Model	Extracts or Specific Components	Antitumor Effect	Biochemical Pathways	References
Pulp	In vitro(cancer cell lines)	Lung adenocarcinoma, cervical cancer, hepatocellular carcinoma	Polysaccharides	Antiproliferative	Cell viability reduction	[22]
				Immunomodulatory	Induction of mouse splenocyte proliferation	
		Gastric cancer, hepatocellular and lung carcinoma	Galactose and mannose	Antiproliferative	Cell viability reduction	[23]
				Antioxidant	Increase in cellular antioxidant activity	
	In vivo(mice)	Chemical-induced liver injury	Pulp extract	Hepatoprotective	Decreased serum ALT and AST levels	[27]
				Antioxidant	Changes in antioxidant enzyme levels	
Peel	In vitro(cancer cell lines)	Hepatocellular carcinoma	Water-soluble crude ethanolic extract	Antiproliferative	Cell viability reduction, clonogenic growth decrease	[75]
				Apoptosis induction	Pre G0/G1 pro-apoptotic peak in cell cycle profile	
		Breast cancer cells	Water-soluble crude ethanolic extract	Antiproliferative	Cell viability reduction, Clonogenic growth decrease	[37]
				Apoptosis induction	Up and down-regulation of gene clusters involved in cell death	
		Breast cancer cells	Specific flavonoid components	Antiproliferative	Cell viability reduction	[36,38]
	In vivo	Murine hepatoma bearing-mice		Inhibition of tumor growth	Reduction in cell proliferation	[75]
		Nude mice bearing human breast infiltrating duct carcinoma	Water-soluble crude ethanolic extract	Tumor mass reduction	Reduction in cell proliferation	[37]
				Apoptosis induction	Caspase-3 activation	
Seed	In vitro(cancer cell lines)	Lung adenocarcinoma, cervical, breast, ovarian cancers and hepatocellular carcinoma	Flavonoid glycosides	Anti proliferative	Cell viability reduction	[51,76]
		Hepatocellular, lung and cervical carcinoma	Sesquiterpene glucosides	Anti proliferative	Cell viability reduction	[77]
		Non-small cell lung cancer	Crude Litchi extract	Anti proliferative	Inhibition of EGF-receptor-pathway	[53]
				Apoptosis induction	Bcl-2 family pro-apoptotic ratio and caspase activation	
		Colorectal carcinoma	Flavonoids and tannins	Anti proliferative	G2/M phase cell cycle arrest with reduction in cyclin levels	[48]
				Apoptosis induction	Increase in Bax level and caspase activation	
		Prostate cancer	N-butyl alcohol extract	Anti proliferative	Clonogenic growth decrease, G1/S phase Cell cycle arrest with increase in p21 and p27 CDK inhibitors	[54]
				Apoptosis induction	Activation of mitochondrial caspase cascade	
				Decrease in cell migration and invasion	Increase in E-cadherin and â-catenin, decrease of vimentin and snail, inhibition of Akt pathway	
		Hepatocellular carcinoma	Semen Litchi containing serum	Anti proliferative	Cell viability reduction	[47]
				Apoptosis induction	Appearance of nuclear morphological features and pre G0/G1 pro-apoptotic peak in cell cycle profile	
	In vivo	mouse xenografts of Ehrlich ascites cells, sarcoma S180 cells, or liver tumor cells	Water extract	Decrease in tumor size		[78]
		hyperplasia of mammary glands rat model	Saponins	Reduction of mammary gland hyperplasia		[49]
		Nude mice xenograft of PC3 cells	n-butyl alcohol extract	Decrease in tumor size		[54]
Oligonol	In vitro	Breast cancer cells	Low MW polyphenols from lychee fruit extract	Apoptosis induction	Modulation of pro-apoptotic Bcl-2 family proteins and MEK/ERK signaling pathway	[73]
	In vivo	DSS-promoted adenoma in the mouse colon		Inhibition of colonic adenoma formation	Reduction of cyclins	[72]
					Variation of oxidative stress markers	
		Melanoma mice models		Inhibition of lung metastasis	Inhibition of lung hexosammine content, and serum sialic acid and gamma glutamyltranspeptidase content	[70]
		Mouse skin and carcinomas and papillomas bearing mice		Suppression of chemically-induced tumorigenesis	NF-êB and C/EBP DNA binding decrease, reduction of ERK 1/2 and P38 kinases, reduction in PCNA and COX2	[71]
